# Local Growth Mediated by Plasmonic Hot Carriers: Chirality from Achiral Nanocrystals Using Circularly Polarized Light

**DOI:** 10.1021/acs.nanolett.1c03503

**Published:** 2021-12-03

**Authors:** Lucas V. Besteiro, Artur Movsesyan, Oscar Ávalos-Ovando, Seunghoon Lee, Emiliano Cortés, Miguel A. Correa-Duarte, Zhiming M. Wang, Alexander O. Govorov

**Affiliations:** † Institute of Fundamental and Frontier Sciences, University of Electronic Science and Technology of China, Chengdu 610054, People’s Republic of China; ‡ Centre Énergie Matériaux et Télécommunications, Institut National de la Recherche Scientifique, Varennes, Québec J3X 1S2, Canada; § CINBIO, Universidade de Vigo, 36310 Vigo, Spain; ∥ Department of Physics and Astronomy and the Nanoscale & Quantum Phenomena Institute, Ohio University, Athens, Ohio 45701, United States; ⊥ Chair in Hybrid Nanosystems, Nanoinstitute Munich, Faculty of Physics, Ludwig-Maximilians-Universität München, 80539 Munich, Germany; ○ Institute for Advanced Study, Chengdu University, Chengdu 610106, China

**Keywords:** Chirality, plasmonics, photogrowth, photocatalysis, hot electrons, nanocrystals

## Abstract

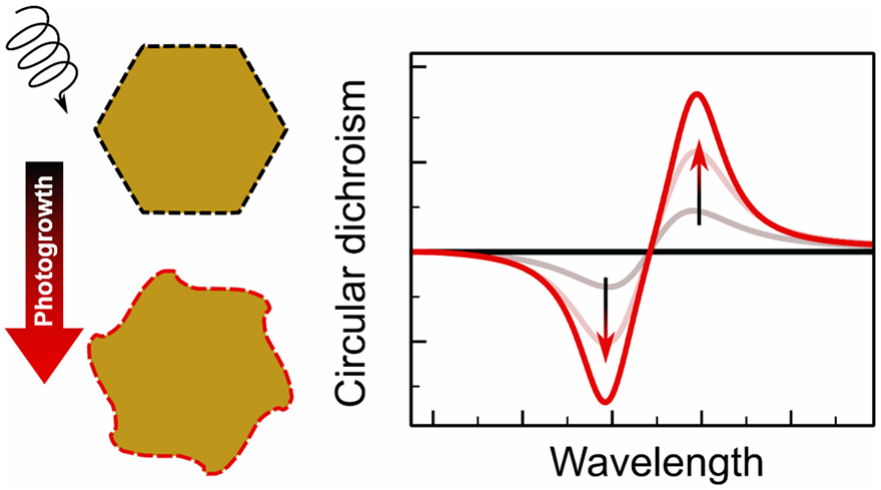

Plasmonic nanocrystals and their assemblies are excellent tools to create functional systems, including systems with strong chiral optical responses. Here we study the possibility of growing chiral plasmonic nanocrystals from strictly nonchiral seeds of different types by using circularly polarized light as the chirality-inducing mechanism. We present a novel theoretical methodology that simulates realistic nonlinear and inhomogeneous photogrowth processes in plasmonic nanocrystals, mediated by the excitation of hot carriers that can drive surface chemistry. We show the strongly anisotropic and chiral growth of oriented nanocrystals with lowered symmetry, with the striking feature that such chiral growth can appear even for nanocrystals with subwavelength sizes. Furthermore, we show that the chiral growth of nanocrystals in solution is fundamentally challenging. This work explores new ways of growing monolithic chiral plasmonic nanostructures and can be useful for the development of plasmonic photocatalysis and fabrication technologies.

Plasmonic nanoparticles support resonant excitations of their mobile electrons under the effect of an external electric field. These plasmonic modes give rise to spectra that are sensitive to the size and geometry of the nanostructure, as these constrain the movement of the quasi-free electrons in the material. Such spectral tunability, together with their strong interaction with light, has made plasmonic nanocrystals (NC) a broadly used tool in nanophotonics, including their use in amplifying and interacting with the chiroptical signature of molecular species.^1,2^ The close connection between plasmonic NCs’ geometry and their optical response makes them especially suitable for creating artificial systems with strong chiroptical responses.^3−7^ These can be fabricated through a variety of advanced fabrication techniques, using either top-down or bottom-up methods^5,8^ to create chiral structures in two^9−14^ and three dimensions^15−17^ or assembling chiral complexes and aggregates in a solution, which is composed of biomolecules and initially nonchiral plasmonic NCs.^18−26^

Plasmonic NCs and their hybrids are also useful as photosensitizing elements in photocatalysis, serving as nanoantennas that concentrate light energy and transfer it to drive chemical transformations.^27−29^ This can be mediated through different mechanisms, with charge transfer events being particularly interesting because they can initiate redox reactions or favor the evolution of a reaction by reducing its activation energy.^30−34^ Charge transfer can occur by either the direct interfacial excitation of a carrier between a metal and its environment^35,36^ or the separate processes of carrier excitation and injection.^37−39^ The diagram in Figure 1a presents this latter mechanism, in which the role of surfaces in the excitation of high-energy excited carriers is very important, because they allow the promotion of an electron within the conduction band by satisfying the constraints of conservation of momentum in the process of photon absorption.^40−43^ Only high-energy excited electrons, to which we will refer as “hot electrons” (HE) hereafter, will be susceptible to leaving the metal. Consequently, when considering the design of plasmonic systems for plasmon-based catalysis, we should consider their capacity for exciting large numbers of hot carriers at the NC’s interfaces.^44^ Moreover, as we will see below, the surface-mediated mechanism for HE excitation will create inhomogeneous excitation rates across the NC’s surface. This also highlights the relevance of plasmonic hot spots in driving the HE excitation, which can arise from interparticle interaction^32,45−47^ or from the geometry^46,48−51^ of metallic resonators.

**Figure 1:**
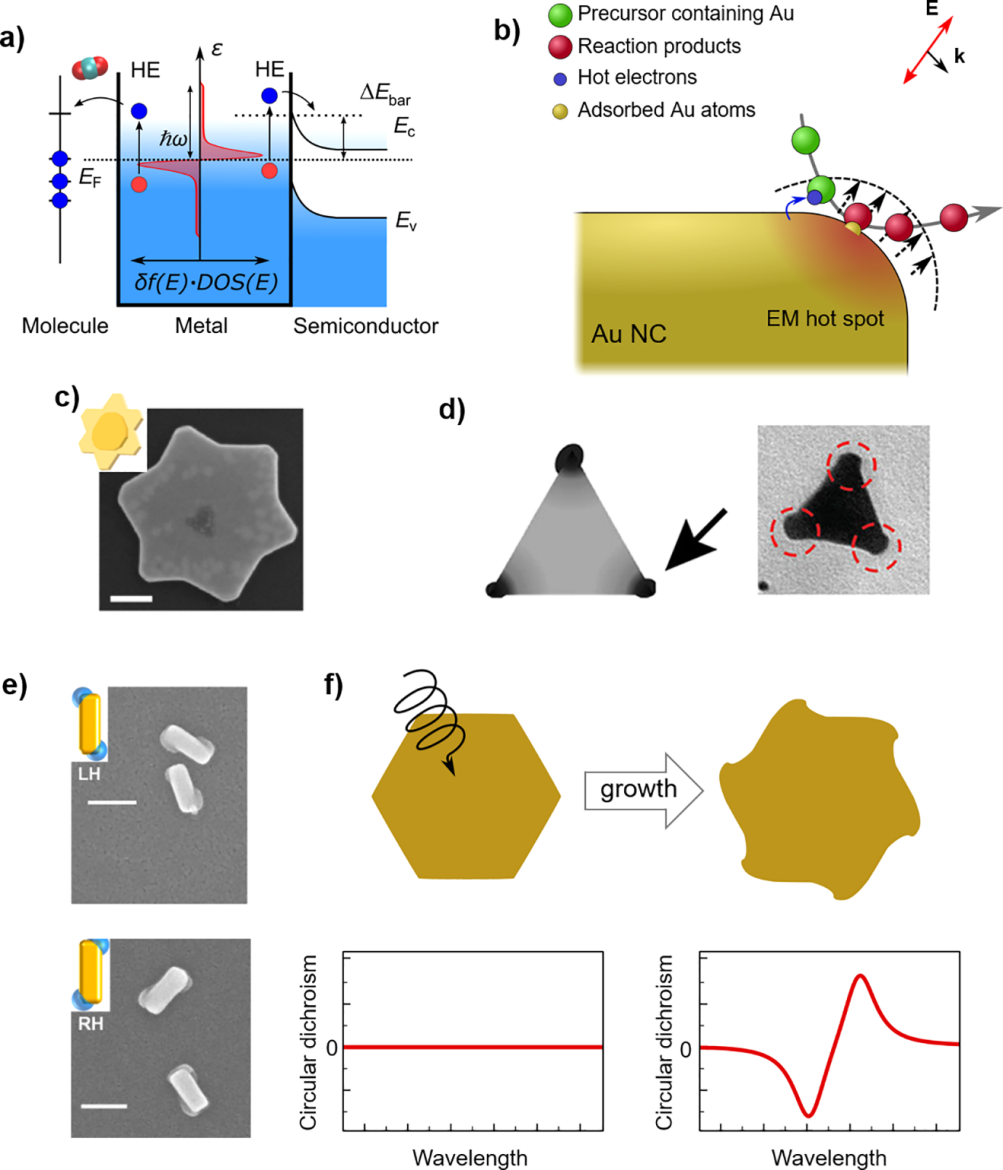
Plasmonic photogrowth mediated by hot electron injection. (a) Schematic diagram depicting hot electron injection from the plasmonic metal to its environment. The electrons need sufficient energy to traverse an interfacial potential barrier or reach empty molecular orbitals. A typical energy distribution of excited carriers in a plasmonic NC (red curve) is overlaid over the metal. (b) Diagram depicting the general mechanism of ion aggregation capable of leading to NC growth. (c) Plasmon-driven anisotropic growth of Au nanostructures: SEM image of the resulting geometry, with a 100 nm scale bar, and inset showing a diagram with initial and final morphologies.^52^ (d) Diagram and TEM image of a silver nanoprism after growth through galvanic replacement at its corners on illumination at its main plasmonic resonance by unpolarized light.^53^ (e) TEM images of Au@PbO_2_ chiral structures successfully photogrown by using circularly polarized illumination over nonchiral Au rods. Scale bars: 100 nm.^54^ (f) Schematic depiction of the development of chirality from a nonchiral NC by using HE-mediated photogrowth under circulary polarized light (CPL). (c) was reproduced with permission from ref (52). Copyright 2020 American Chemical Society. (d) was reproduced with permission from ref (53). Copyright 2020 American Chemical Society. (e) was reproduced with permission from ref (54). Copyright 2018 American Chemical Society.

## Photoinduced Growth and Chirality

Among the processes that can be driven by the excitation of plasmonic hot carriers, some can change the plasmonic NC itself, as schematically depicted in Figure 1b. These include growth by aggregation of metal ions,^53,55−58^ shrinkage by photoetching,^52,55^ and growth or accretion of other materials on its surface.^59−61^ The schematic diagram in Figure 1b illustrates the fundamental idea behind the HE-directed metal growth, which will depend on the local rate of HE injection and leads to locally induced growth, changing the NC’s shape. This picture of inhomogeneous HE-directed growth contrasts, for instance, with the resulting surface pattern expected from both growth^62^ and surface reactions^63,64^ driven by photoheating. The latter should instead be nearly homogeneous at the plasmonic NC’s surface, a consequence of the large thermal conductivity of the metal.^64^ It is worth noting, however, that carrier-mediated growth can also develop with homogeneous patterns, especially when it depends on bulk-type interband transitions.^65^ Inhomogeneous growth can also be the consequence of distinct reaction probabilities on different sites, such as the flat nanostars depicted in Figure 1c, whose final shape arises from the balance of electron and hole injection at different crystalline faces,^52^ or can be controlled with the curvature of the edges in the NC geometry.^66^ Also, it can arise from the larger excitation rates of hot carriers at plasmonic hot spots, producing growth patterns centered on these regions, such as the example in Figure 1d showing the results of the galvanic reduction of silver triangular nanoprisms on excitation at their plasmonic resonance.^53^ Importantly, the growth pattern can also reflect the symmetry of the incoming illumination, such as that shown in Figure 1e, where the chiral deposition of PbO_2_ over nonchiral Au rods is achieved by illuminating them with circularly polarized light (CPL).^54^ This experiment exemplifies a category of cases where a photocatalytic process can transform achiral plasmonic NCs in chiral systems when they are illuminated with circularly polarized light. Such a chiral growth process, schematically illustrated in Figure 1f, will be the focus of our study in this letter, as we explore it using a novel theoretical approach that explicitly models the inhomogeneous deformations in the geometry of Au NCs caused by their local HE injection rates. After discussing the general properties of the process using the results of several model geometries, we will show how our algorithm reproduces the main features of the experimental growth in ref (54) (Figure 1e), which we use as a benchmark to support the validity of our approach.

**Figure 2:**
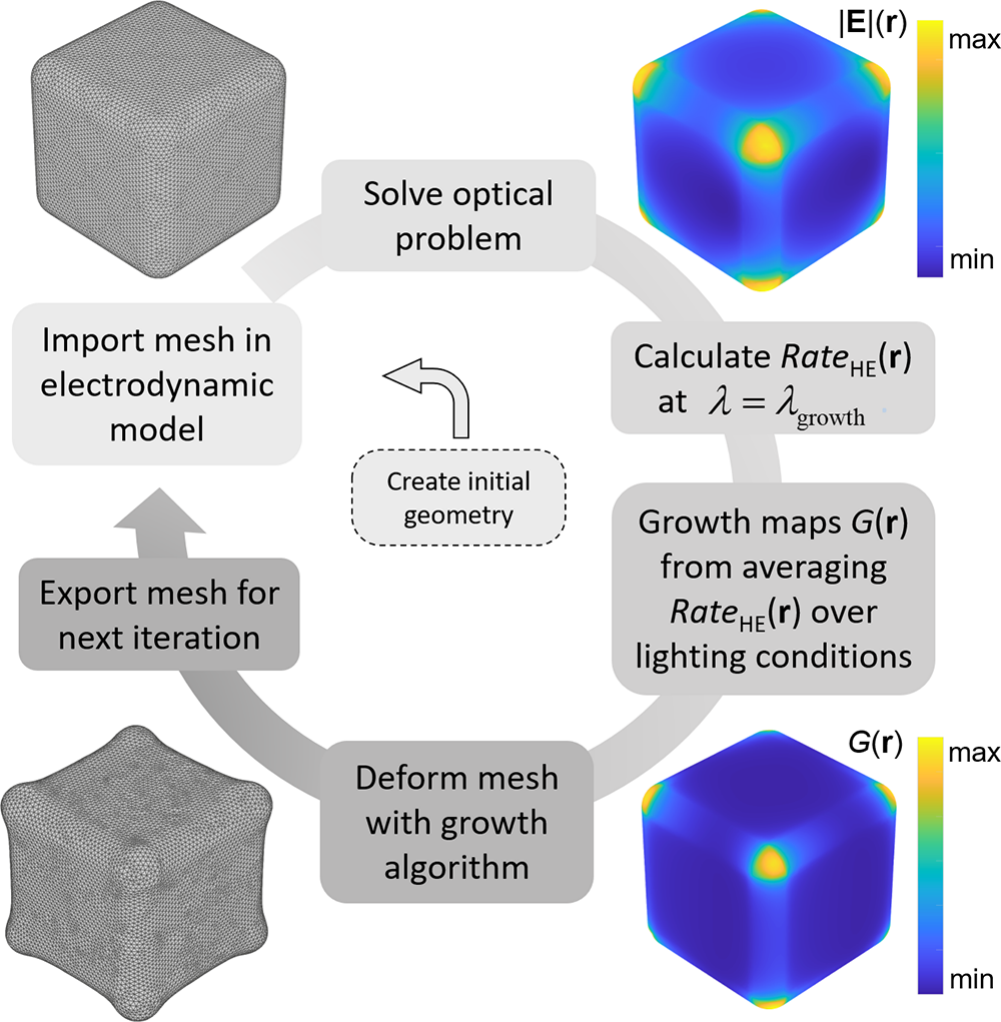
Scheme of the main steps in the computational growth algorithm. Once a given geometry is imported into the solver, we can obtain its spectral response and, importantly, calculate the maps of intraband hot carrier excitation that will inform the growth maps. Once we have these data for all relevant illumination conditions, we obtain the growth maps and deform the mesh of the NC following these data. At that point we have a new geometry that can be used to initiate a new iteration of the photogrowth cycle. Consequently, this photogrowth algorithm instantiates a nonlinear growth model in which the optical response of the NC changes as its surface grows, thus affecting the growth pattern in each subsequent step.

This study connects with previous work on the usage of chiral plasmonic NCs in photocatalysis, offering novel possibilities for controlling the evolution of plasmon-driven chemical reactions. The difference in amplitude at the resonances between the enantiomers results in a net circular dichroism (CD) over the maximum reaction rates that each can cause through HE injection. In ref (67) we have explored this idea in the context of the differential promotion of the growth of two enantiomers of colloidal Au helices under CPL. Using comparatively simple growth models, we were able to illustrate how a racemic mixture of these nanoantennas would develop into an optically active solution through this CPL-sensitive growth.^67^ In this letter, we present a more sophisticated growth model that accounts for the nonhomogenous excitation of HE carriers across the NC’s surface, allowing us to show how nonchiral nanostructures can develop chirality through CPL-induced growth. The combined system of a nonchiral plasmonic NC and impinging CPL presents a local electromagnetic chirality^68^ that can function as the seed for a subsequent chiral growth process. Our photogrowth algorithm, sketched in Figure 2 and whose details can be found in the Supporting Information, iteratively deforms the mesh of the NC as a function of the local rate of HE excitation, computed from numerical electrodynamic results following a previously reported formalism^42^ as
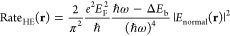
where Δ*E*_b_ is the energy difference between the acceptor state–or the top of the Schottky barrier–with respect to the Fermi energy of the metal, *E*_F_. The energy of the impinging photons is *ℏω* and, crucially, the excitation rate depends on the component of the electric field normal to the NC surface. The rates of excitation of high-energy carriers will be averaged over the response of the NC under different directions of the light’s incidence depending, as described below, on the simulated illumination conditions. These values produce growth maps that will inform the deformation of the NC’s mesh, a process that creates the initial input of another step of this iterative cycle.

## Computational Chiral Growth: Plasmonic Cube and Illumination Conditions

**Figure 3:**
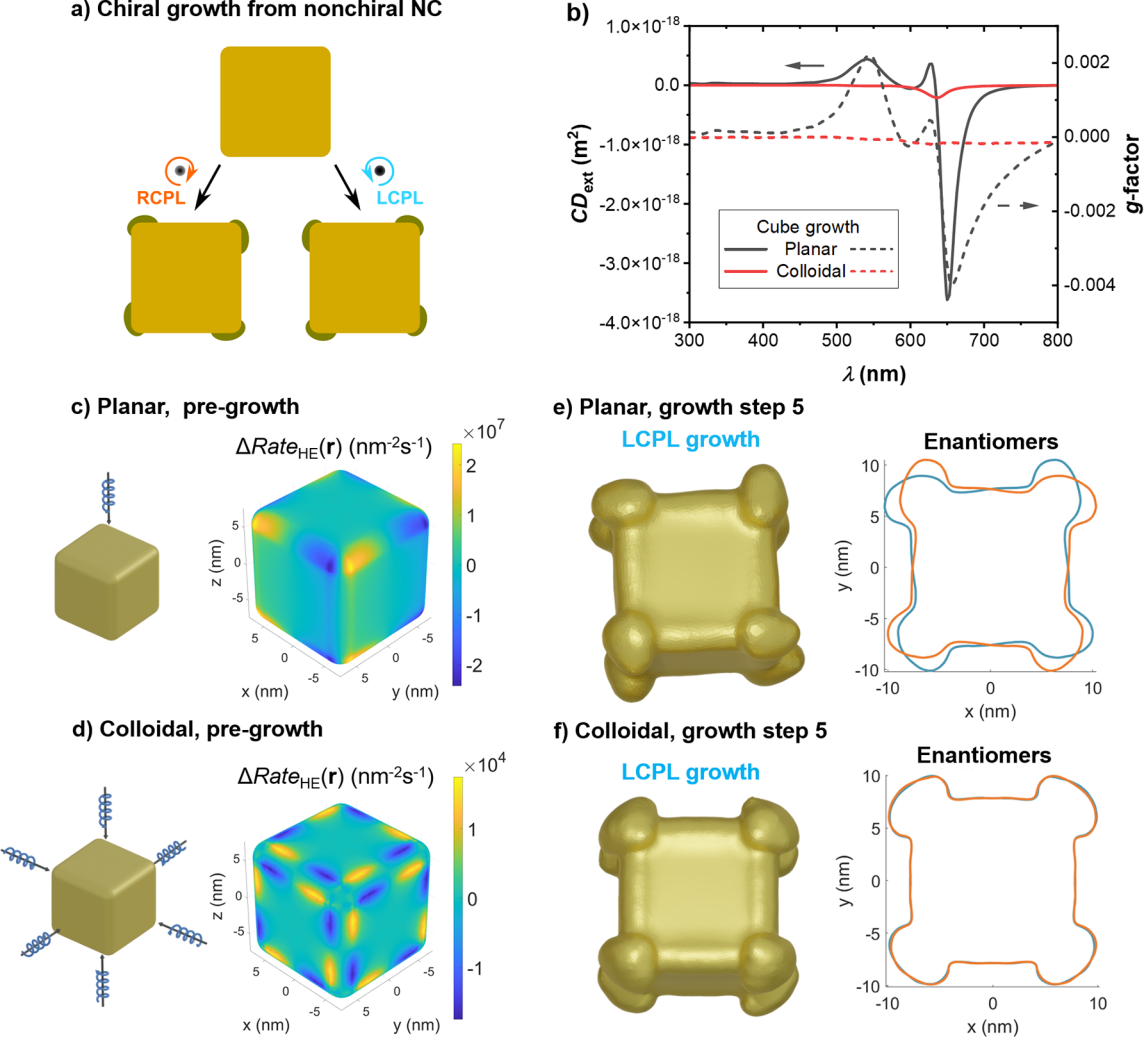
(a) Schematic diagram for the chiral growth from a nonchiral Au cube, with the polarization of light controlling the chirality of the final structure. (b) Comparison of the chiral signal of a Au cube grown under planar and colloidal conditions, after five growth steps. The solid lines represent the circular dichroism of their extinction cross sections, while the dashed lines are their dissymmetry factors, or *g*-factors. (c, d) Diagrams of the planar (colloidal) illumination conditions and maps of the differential rate of HE excitation under both polarizations of CPL. (e, f) Mesh geometries of the cube after five steps of growth under left circularly polarized light (LCPL) for planar and colloidal conditions, next to a comparison with its enantiomer using a cross section of both geometries at a *z* plane cutting them 7 nm above their centers.

Let us first examine in some detail the case of a Au cube (with sides of 15 nm and edges rounded with a 2 nm radius), for which we can see a summary of results in Figure 3. This is a simple, small nonchiral geometry that has the interesting property, in the context of photoinduced catalysis, of presenting electromagnetic hot spots at its vertices. These lead to faster generation rates of hot electrons, which can in turn drive the metal accretion process. Furthermore, the flat faces of the cube provide features at which small chiral variations can take hold and grow (Figure 3a). From this, we should in general expect that photogrowth induced by local HE injection under CPL could produce shapes with some chirality, albeit small. However, the illumination conditions will be crucial in determining the potential magnitude of such chiral growth. We can contrast the growth of a cube under a CPL propagated under a single direction, equivalent to a NC deposited over a substrate and illuminated with a beam impinging orthogonally (*planar* illumination condition), with that of the averaged response of three pairs of counterpropagating CPL beams, equivalent to the conditions experienced by a colloidally suspended NC (*colloidal* illumination condition). The chiral signals resulting of five steps of growth under each of these conditions are shown in Figure 3b, where we can clearly see that the planar growth conditions are more conducive to generating new geometries producing chiral signals, with the CD_ext_ and *g*-factor being larger by more than one order of magnitude for the results of planar growth with respect to those of colloidal growth, even though the latter are strictly nonzero.

**Figure 4:**
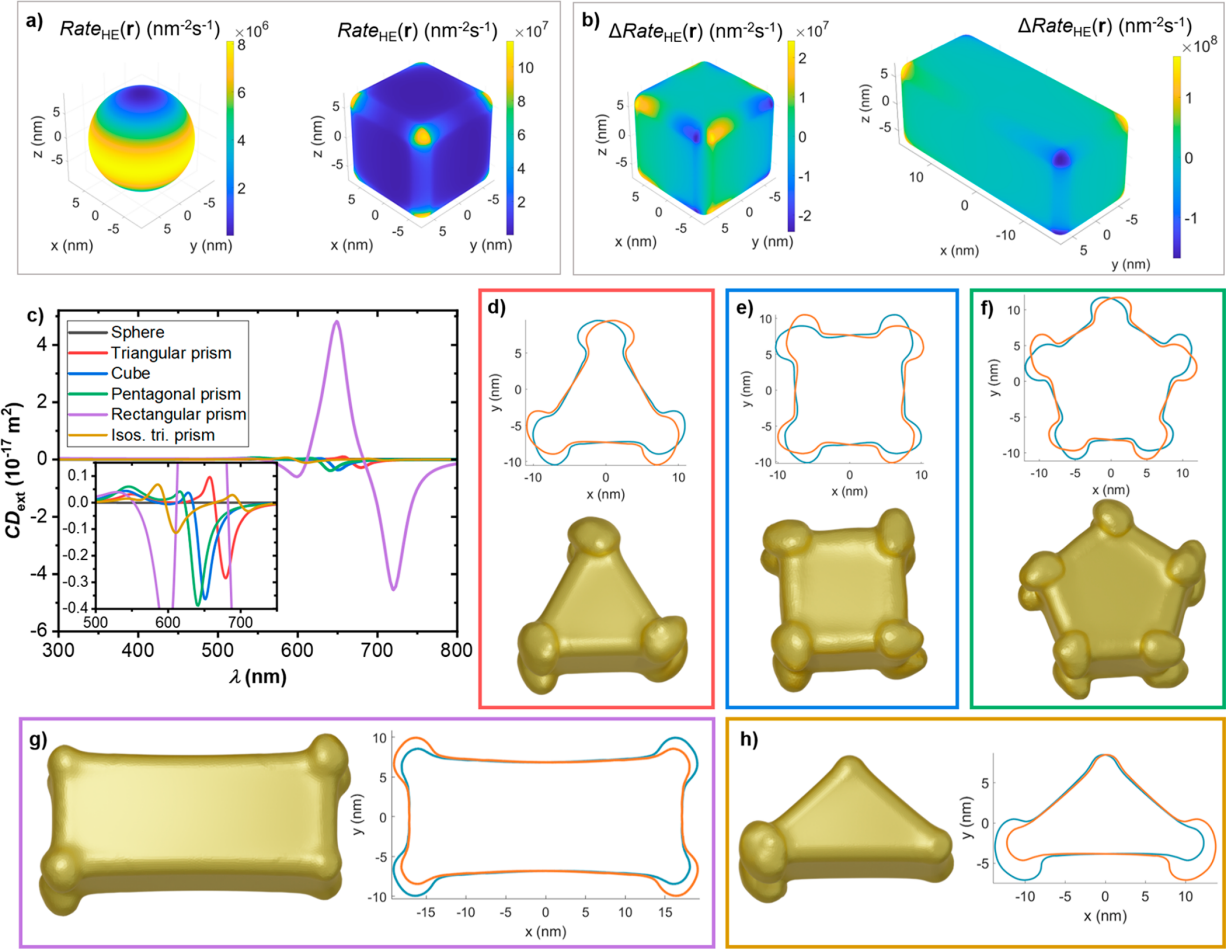
Initial Rate_HE_(**r**) maps for small nonchiral Au geometries under planar illumination conditions, immediately prior to the first growth step. Each NC is illuminated at its main plasmonic resonance (see Figure S8). (a) Sphere and cube under planar LCPL. The rotational symmetry of the sphere does not give rise to any discrete hot spot, while the sharper features of the cube provide locations for the stronger field enhancement. (b) Map of ΔRate_HE_(**r**) for the cube, a representative geometry with sharp features inducing an asymmetry between LCPL and RCPL, and a rectangular prism. Increasing the aspect ratio of the geometry above 1, i.e. elongating the NCs, creates a much stronger asymmetry between LCPL and RCPL. Each polarization preferentially excites a set of corners of the prism. Also note the order of magnitude difference with respect to the cube’s data. The panels below explore the resulting geometries after five steps of iterative growth under LCPL for planar illumination conditions. (c) Extinction CD spectra for the photogrown sphere and the geometries in other panels. (d–h) Resulting geometries after five steps of growth under planar illumination at the main plasmonic mode of a range of NC geometries (see also Figure S5), with a cross-sectional cut of each geometry and its enantiomer to evaluate the degree of their chirality. The original shapes are a triangular prism (d), a cube (e), a pentagonal prism (f), a rectangular prism (g), and an isosceles triangular prism (h). The growth pattern of the sphere can be found in Figure S1. The growth pattern followed by the rotationally symmetric NCs is perturbed when we introduce a longer side and further reduce the rotational symmetry of the system by considering the rectangular prism or the isosceles triangular prism. These latter geometries produce more immediately apparent chiral features upon photogrowth.

In order to find the origin of such disparate behavior, it is useful to look into the initial response of the still-nonchiral geometry to these two illumination conditions, before the first growth step is undertaken. The development of chiral features in our initially nonchiral cube proceeds progressively. However, it is clear that the initial step is of singular significance, as the NC has to transition from zero to a small but nonzero chirality if the overall growth is to accrue an overall chiral development. This can occur, of course, due to the chirality introduced in the system through the incoming CPL. In Figure 3c,d we can see, as a measure of the initial chirality of the system composed by the NC and the impinging radiation, surface maps of the difference between the local rate of HE excitation under opposite polarizations of CPL:
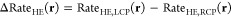
There are two major points to note in the maps in Figure 3c,d. The first is the difference in the patterns produced at the surface of the two NCs, even though under both illumination conditions the differential features of HE excitation are concentrated around the vertices of the cube, as we would expect because of the presence of hot spots at those locations (see also Figure 4). In both cases the corners break the rotational symmetry of the CPL, but the asymmetry under the planar illumination condition simply breaks into the four-fold rotational symmetry of a square, while under colloidal illumination the same reduction occurs on the three main axes of the cube. Furthermore, the pattern is now antisymmetric along these axes due to light propagating across both directions of a given axis. The second major distinction is the difference in absolute magnitude in ΔRate_HE_ between the two illumination conditions of approximately three orders of magnitude. Thus, any chiral feature arising through photogrowth in a colloidal suspension might be dwarfed by other factors introducing variability, such as initial irregularities in the geometry, gradients in the local conditions in solution, or departures from an ideally homogeneous isotropic illumination of the NC. The resulting photogrown geometries in Figure 3e,f show that only planar illumination produces features that are clearly chiral, whereas the two enantiomers resulting from colloidal illumination conditions present an almost perfect overlap. The chiroptical signal resulting from the nonlinear growth process under colloidal conditions is nonetheless nonzero, as shown in Figure 3b, but it is smaller than that resulting from planar illumination by more than one order of magnitude.

## Relevance of Seed Geometry in Chiral Growth

Let us now contrast the response of several Au nonchiral geometries to planar illumination conditions. The geometries that we will consider consist of a sphere, a triangular prism with three equal sides, the aforementioned cube, a pentagonal prism, a rectangular prism, and a triangular prism with a longer side. The first four systems have similar volumes, the rectangular prism shares the height of the other prisms, 15 nm, and the last triangular prism shares the length of its short sides with the equilateral triangular prism. Before looking at the geometries resulting from subjecting these NCs to planar chiral illumination, we can examine their initial plasmonic responses: i.e., prior to the first iteration of the growth algorithm taking place. To do this, in Figure 4 we showcase the local maps of Rate_HE_(**r**) or ΔRate_HE_(**r**), which will determine growth rates and affect the differential growth under each polarization, respectively, in the first growth step. The first thing we should note is that the presence of edges and corners not only introduces strong hot spots, as we can see in the comparison between a sphere and a cube (Figure 4a), but also breaks the perfect rotational symmetry of the spherical NC (or, for that matter, cylindrical). This feature allows the combined system of NC and CPL to show chiral features in the form of the distribution of HE excitation across the NC’s surface, even though its geometry is fully nonchiral. In contrast, a perfect sphere does not provide adequate initial conditions to initiate chiral growth, as the combination of the geometry and an impinging beam of CPL preserves the rotational symmetry and thus further changes in geometry should be rotationally symmetric as well.^7^ Once we consider geometries with broken rotation symmetry, it is useful to contemplate the progressive reduction of such symmetry. The Supporting Information contains detailed ΔRate_HE_(**r**) maps for five polyhedra with planar cross sections belonging to the *C*_1*v*_, *C*_2*v*_, *C*_3*v*_, *C*_4*v*_, and *C*_5*v*_ point groups, alongside a discussion of the trends observed across them. Here we will highlight the sharp distinction appearing when considering systems with *C*_1*v*_ and *C*_2*v*_ symmetries. In the comparison presented in Figure 4b, we can contrast the chiral features appearing at each of the vertices of the cube with those appearing on alternating vertices on the rectangular prism. From this difference, we can expect a clearer resulting chiral geometry arising from the photogrowth of the rectangular prism under CPL, and this property will be replicated by the isosceles triangle with *C*_1*v*_ symmetry (Figure S2).

**Figure 5:**
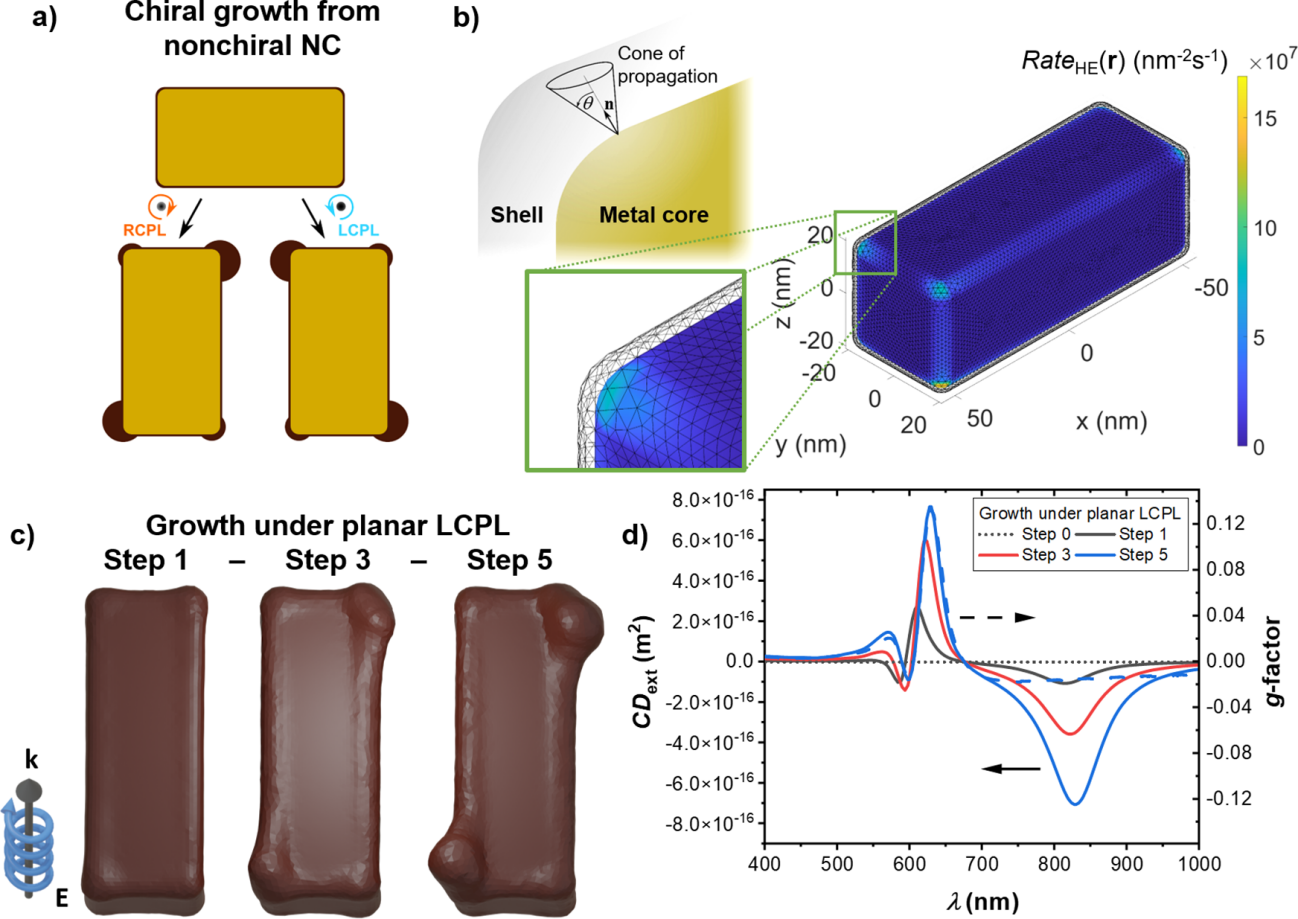
Chiral photogrowth of a Au@PbO_2_ rectangular prism. (a) Diagram depicting the chiral growth process (light moving into the page). In this case only the surrounding PbO_2_ layer is modified, and the Au rectangular prism keeps its shape. Our model begins with a thin (1 nm) nonchiral layer of PbO_2_ covering the metal, modeled as a dielectric with a refractive index of *n* = 2.3.^69^ (b) Model of the Au@PbO_2_ system, showcasing the Rate_HE_(**r**) at the interface of the two materials under λ = 550 nm illumination. The outer mesh corresponds to the outer surface of the PbO_2_ layer. The inset diagram depicts the cone, with an opening angle of 2θ = 120°, that propagates from each point at the Au NC’s surface the contribution from carriers excited at the metal–semiconductor interface toward the growth rates of the PbO_2_ area inside the projected cone. (c) Models of the Au@PbO_2_ prism after one, three, and five growth steps under LCPL with λ = 550 nm, under planar illumination conditions. The models are seen from below (light moving out of the paper), to highlight the pattern developed on this side of the NC. (d) Extinction circular dichroism (solid and dotted lines) and *g*-factor (dashed curve) of the prism at different growth stages.

The initial chiral response in the pattern of excitation of hot carriers for the nonchiral NCs, illustrated in Figure 4a,b and Figure S2, will seed the geometries produced through photogrowth. In Figure 5 we show these results for the NCs breaking the continuous rotational symmetry, after five iterations of the HE-directed growth mechanism under planar illumination conditions (Figure S3 showcases the intermediate geometries). We are focusing on planar illumination because photogrowth in a colloidal scenario does not lead to meaningfully chiral systems. Results under both illumination conditions can nonetheless be found in Figures S4 and S5. From the CD data in Figure 4c, it is clear that the resulting chirality of the rectangular prism is well above that of the smaller NCs, due to its distinct chiral pattern and, importantly, its larger aspect ratio and consequent spectral sensibility of its longitudinal plasmonic mode. The final shapes of the originally nonchiral seeds are in accord with our expectation from examination of the initial Rate_HE_(**r**) maps. We can see the chirality developing through the directional elongation of the photogrown features for the first three geometries in Figure 4d–f. Further remarks on these NCs and their chiroptical signal can be found in the Supporting Information. Then, we see in Figure 4g how the asymmetric growth manifests itself with the growth of large features at only two pairs of corners of the rectangular prism. This pattern is further reduced for the isosceles triangular prism, for which we see in Figure 4h a single edge showcasing prominent growth. Thus, the four geometries develop a number of features that are in accord with the *C*_*nv*_ point symmetry of each of their planar cross sections, with each NC showcasing significant growth at each of the symmetrically unique features that reduce the symmetry of the system from a continuous rotational symmetry. It is relevant to note that we could keep reducing the symmetry of the geometries presented here by contemplating, for instance, a prism with a scalene triangle in its cross section (see Figure S6). Such a seed would be different in that it is intrinsically chiral in 2D, whereas all the previous seeds were strictly nonchiral.

## Chiral Growth of Hybrid NCs

We will now discuss one last system introducing a few variations. We will again consider a rectangular prism, the best geometry to generate a chiral shape among those considered above, but now with a larger size of 100 × 40 × 40 nm^3^. Also, even though we will still model a Au prism, in this case we will consider that the HE-mediated growth mechanism is the accretion of a dielectric layer on top of the metal, as depicted in Figure 5a. This is a model created after the experimental work by Saito et al.,^54^ who grew PbO_2_ structures over rectangular Au prisms deposited over a substrate. Using CPL, this growth process leads to significantly chiral NCs (see Figure 1e), and with our numerical model we can show how this optical process can, from nonchiral particles, lead to the final chiral geometries observed experimentally.^54^ Given that we are now considering the growth of a second material over the surface of the Au NC, an extended model (Figure 5b) makes use of a supporting hypothesis, namely that the propagation of hot carriers injected into the semiconducting shell will occur mainly along a straight line.

As in the experimental work,^54^ we will consider photogrowth under LCPL at λ = 550 nm, thus targeting a high-energy resonance different from the main plasmonic mode (see Figure S9 for details about this prism’s spectrum and the symmetries of its two plasmonic modes), with the main features appearing in this case most notably at the bottom of the NC. This can be seen more clearly in Figure S9 but is also seen in examining the changes in shape with the photogrowth shown in Figure 5c, where the panel illustrates the bottom of the NCs, as defined by the propagation of light. In this sequence of models, we can see the growth ofthe PbO_2_ shell around the Au prism as it develops a clear chiral pattern. We can also appreciate how, after several photogrowth steps, the lumps of semiconductor start to extend along the long side of the prism, thus paralleling the observed shape of the experimental chiral systems grown by Saito et al. (see Figure 1e).^54^ Finally, we can observe in Figure 5d that the spectral profile of the photogrown Au@PbO_2_ systems shows significantly stronger chiral signals in comparison to those developed by the smaller NCs, including the small Au rectangular prism. Nonetheless, the *g*-factors of the small Au prism (Figure S7) and the Au@PbO_2_ prism (Figure 5c) are of comparable magnitude, thus indicating that their chiroptical signal relative to their extinction is similar. Moreover, we can see that the overall features developed in the chiral signals of the NCs match those observed in the experimental work of Saito et al. in ref (54), presenting further evidence that our algorithmic approach to model the HE-directed growth of the PbO_2_ shell reproduces well the experimental results. In the CD_ext_ spectra Figure 5d, we can observe the increasing magnitude of two main chiroptical features, a bisignate signal arising close to the growth wavelength and an additional chiral signal present at the long-wavelength plasmonic resonance of the system. At the same time, the *g*-factor spectrum in the same panel indicates that the long-wavelength feature in CD_ext_ is much smaller than the average extinction between the enantiomers. The Supporting Information also contains a depiction of the geometry of these Au@PbO_2_ after an equivalent process of photogrowth under colloidal illumination conditions (see Figure S10).

In summary, we have presented a novel algorithmic approach for modeling the inhomogeneous growth of nanostructures, as mediated by the local excitation of high-energy hot electrons under optical excitation. With it, we have studied the effect that circularly polarized light can have on the growth patterns of different nonchiral geometries. We have explored the relevance of their shape toward the development of chiral features and contrasted two photogrowth scenarios connected with realistic setups, having the NCs either deposited over a substrate or dispersed in a solution. Interestingly, our computational model, which has successfully replicated the main properties of a notable experimental report on the optically induced chiral growth over a large Au prism,^54^ also shows that a comparable effect should be achievable when illuminating NCs with sizes significantly smaller than their resonant wavelength. It has also been demonstrated that the growth of chiral NCs with CPL in solution is fundamentally challenging for both small and large NC sizes, due to the averaging effect of the random orientations of the NC with respect to the source of light. This points to the need for further investigation to achieve significantly chiral shapes under this type of chiral photogrowth. Potential avenues for increasing the chiral photogrowth in colloidal conditions include using NC seeds with specific asymmetries, surface inhomogeneities, external sources of symmetry-breaking such as particle–particle interaction, or even methods that speed up the growth reaction relative to the random drift of the NCs. In more general terms, our results demonstrate the potential of this novel way of studying and predicting particle photogrowth and photochemistry, which could be readily generalized to consider different physical processes as their driving force. Thus, this algorithmic modeling approach could predict and contrast the reshaping driven by different physical mechanisms or in processes other than growth such as melting and etching. Further developments can also study such scenarios in more complex systems, such as plasmonic complexes with NCs in close interaction or complex metamaterials supported over a substrate.

## Supporting Information Available

Detailed theoretical framework and computational methods, photogrowth of Au sphere, differential response maps of different NCs, images of the iterative growth of the different geometries, comparison of cross sections of chiral enantiomers, extended optical data of NCs, and characterization of short- and long-wavelength resonances in a Au@PbO_2_ prism (PDF)
