# Supplementary material for: Local Growth Mediated by Plasmonic Hot Carriers: Chirality from Achiral Nanocrystals Using Circularly Polarized Light
Source: Nano Lett. 2021 Dec 3;21(24):10315–24. doi: 10.1021/acs.nanolett.1c03503 (PMC8704195; doi:10.1021/acs.nanolett.1c03503)
Supplement: Supplementary file 1 — nl1c03503_si_001.pdf [file nl1c03503_si_001.pdf]

SUPLEMENTARY INFORMATION

Local Growth Mediated by Plasmonic Hot Carriers: Chirality from Achiral Nanocrystals using Circularly Polarized Light

Lucas V. Besteiro[1,2,3,*], Artur Movsesyan[1,4], Oscar Ávalos-Ovando[4], Seunghoon Lee[5], Emiliano Cortés[5], Miguel A. Correa-Duarte[3], Zhiming M. Wang[1,6,*], Alexander O. Govorov[1,4,*]

1 Institute of Fundamental and Frontier Sciences, University of Electronic Science and Technology of China, Chengdu, 610054, People's Republic of China

2 Centre Énergie Matériaux et Télécommunications, Institut National de la Recherche Scientifique, Varennes, Québec J3X 1S2, Canada

3 CINBIO, Universidade de Vigo, 36310 Vigo, Spain

4 Department of Physics and Astronomy and the Nanoscale & Quantum Phenomena Institute, Ohio University, Athens, Ohio 45701, United States

5 Chair in Hybrid Nanosystems, Nanoinstitute Munich, Faculty of Physics, Ludwig-Maximilians-Universität München, 80539 Munich, Germany

6 Institute for Advanced Study, Chengdu University, Chengdu 610106, People's Republic of China

* Email address: lucas.v.besteiro@uvigo.es, zhmwang@uestc.edu.cn, govorov@ohio.edu

Computational methods

The results presented in this letter were obtained with a photogrowth algorithm describing a process depending on the injection of interband hot carriers at the plasmonic NC's surface. As detailed below, we posit a growth process in which the local size increase depends linearly on the local rate of hot carrier injection, evolving through the illumination of plasmonic NCs surrounded by suitable reactants. Our model considers discrete time steps, so that we can update the optical response of the NC after its shape changes slightly, and thus account for the intrinsic nonlinearity of the growth process. Herein, we will concern ourselves with two different scenarios, where gold nanocrystals either grow or promote the accrual of a semiconductor layer over them. The model for both is conceptually similar, but in the former there is a more marked nonlinearity in the growth process, as the shape of the metal resonator changes after each time step. In both cases, we will consider that the only energy transfer mechanism by which the plasmonic nanoparticles contribute to these chemical reactions sustaining the NCs' growth is the indirect injection of hot electrons. Importantly, by modelling purely HE-induced photogrowth, our results present a benchmark result that can be used to determine whether an observed photocatalytic mechanism is spatially localized, as with the injection of locally-excided HE, or instead non-spatially resolvable within a NC, as e.g. temperature-driven catalysis with good thermal conductors such as gold. This is so

because the high thermal conductivity strongly smooths, to the point of practically eliminate, the spatial inhomogeneities on the NC's temperature in the steady state,[1,2] thus leading us to expect that a temperature-directed growth will not show a different effect at the NC's hot spots. Thus, absent a significant delocalization of the excited plasmonic hot electrons,[3] one should expect that NC growth mediated by hot electron injection will privilege growth at plasmonic hot spots, which is precisely what we observe in our present model.

A critical part of our growth model consists in computing the spatial distribution of hot carrier excitation at the NC's surface, as this will be the fundamental factor driving the geometrical change in the photogrowth. This is one of the few mechanisms allowing the excitation in plasmonic materials of electrons with high energies,[4,5] sufficient to traverse the potential barrier of the metal-environment interface, and which can be of particular relevance because it ensures that these carriers are actually excited close to that interface. The diagram in Fig. 1 also contains the sketch of a typical distribution of excited carriers in the steady state of a plasmonic nanoparticle under illumination,[6–8] where most of the excited carriers have energies below the threshold imposed by the interfacial potential barriers.[9] The dephasing of the collective plasmonic mode produces in general a large number of low-energy carriers, close to the Fermi level, while the excitation of high-energy carriers is limited by conservation of momentum.[9,10]

We will solve the electrodynamic problem posed by a plasmonic NC irradiated by a plane wave, and obtain from these data the spatial maps for the production of hot electrons. We do this by computing the expected local rates of hot electron excitation arising from the breaking of translation symmetry by the NC's surface, which allows local electronic states to absorb the full energy of an incoming photon.[9] Generalizing from a full quantum formalism, we have developed a useful model that allows the calculation of the power directed to the high-energy hot electrons for arbitrary geometries.[11–13] At the core of this approach is the following equation, of which a detailed account of its derivation can be found in Ref. [7]:

$$Rate_{\mathrm{HE}} = \frac{2}{\pi^2}\frac{e^2 E_{\mathrm{F}}^2}{\hbar}\frac{\hbar\omega - \Delta E_{\mathrm{b}}}{\left(\hbar\omega\right)^4}\int_{S_{\mathrm{NC}}} \left|E_{\mathrm{normal}}\left(\mathbf{r}\right)\right|^2 \mathrm{ds} \qquad \text{(S1)}$$

With it, we can obtain the rates of excitation of high-energy intraband electrons and holes at the surface of plasmonic nanosystems of arbitrary sizes and geometries, as driven by the electric field at the surface. This information is obtained by obtaining the classical electrodynamical response of the plasmonic nanoparticle computationally, which enters the equation through E_{normal}, the component of the electric field normal to the surface of the particle and inside the metal. The equation also includes the energy difference between the metal's Fermi energy, E_{F}, and the acceptor state or the top of the Schottky barrier, ΔE_{b} (see Fig. 1a in the main text), as well as the energy of the incoming photon, $\hbar\omega$. One should note that Eq. S1 above is used to calculate the total rate of hot electron excitation per NC, but by eschewing the surface integral over the NC's surface, S_{NC}, we can obtain a $Rate_{\mathrm{HE}}\left(\mathbf{r}\right)$ that provides us with the spatial distribution of the surface-assisted hot electron excitation:

$$Rate_{\mathrm{HE}}\left(\mathbf{r}\right)=\frac{2}{\pi^2}\frac{e^2E_{\mathrm{F}}^2}{\hbar}\frac{\hbar\omega-\Delta E_{\mathrm{b}}}{\left(\hbar\omega\right)^4}\left|E_{\mathrm{normal}}\left(\mathbf{r}\right)\right|^2\,\mathrm{ds} \quad \text{(S2)}$$

Then, the methodology followed to study the locally-induced HE growth will rely on the algorithmic modification of the 3D mesh defining the metal NC, and proceeds in an iterative way. Fig. 2 sketches the overall algorithm by highlighting the central tasks performed at each step. Each full iteration of the algorithm consists on the (i) calculation of the optical response of the plasmonic NC at the wavelength of the source light, (ii) the extraction of HE excitation maps from that data, (iii) the deformation of the NC's geometry according to the HE density maps, and (iv) computing the full optical spectrum of the resulting NC's geometry. A full iteration thus calculates the changes that the external illumination operates on the NC over a short period of time, with no explicitly defined length. As each growth step changes the shape of the optical resonator, it is clear that this growth algorithm is, similarly to the real phenomenon, a strongly nonlinear process where the changes in the system affect its subsequent response to the impinging light.

More specifically, we first perform a full electrodynamic simulation of the optical response of a given gold geometry, using the commercial software package COMSOL Multiphysics, which implements solvers in the framework of Finite Element Methods. The electric field values obtained as solutions will be used to calculate $Rate_{\mathrm{HE}}\left(\mathbf{r}\right)$ using Eq. S2. Each of these simulations are performed at specific illumination conditions, choosing the source of light as having the wavelength at which the non-chiral geometry has peak in $Rate_{\mathrm{HE}}$ (Eq. S1) and a given polarization of light. Moreover, we will contemplate two distinct types of illumination conditions, chosen to mimic experimental scenarios. The first condition models the growth of NCs deposited over an inert substrate and with the source of light impinging directly over them, while the second condition models the growth of NCs in a colloidal suspension and involves the averaging of the local rates of generation of HE over six directions of incidence along the three Cartesian axes. We will refer to these growth conditions as *planar* and *colloidal*, respectively. Implicit in the methodology followed for the colloidal condition is the assumption that the kinetics of the reaction will be slow in comparison with the typical timescales for the stochastic movements of the NCs. In both growth conditions, we will report circular dichroism data obtained by proving the system in the same illumination conditions as the growth.

We will mainly discuss two magnitudes quantifying the chiral properties of the NCs. Firstly, focusing on the direct observation of the differential response of a system to left- and right-CPL. This is quantified by the circular dichroism (CD) of the extinction of a sample, which is simply the difference between its extinction evaluated under LCP and RCP light. If expressed in terms of the extinction cross sections of the NC, this is simply

$$CD_{\mathrm{ext}}=\sigma_{\mathrm{ext}}^{\mathrm{LCP}}-\sigma_{\mathrm{ext}}^{\mathrm{RCP}} \quad \text{(S3)}$$

Another useful measure to quantify the degree of optical chirality of a sample is the dissymmetry factor *g*, or g-factor, which is a normalized value that facilitates the comparison

between the degree of chirality of NCs with different absolute cross sections. It is defined as the ratio of CD_{ext} over the extinction of the sample averaged over both polarizations of CPL,

$$g = \frac{\sigma_{\mathrm{ext}}^{\mathrm{LCP}} - \sigma_{\mathrm{ext}}^{\mathrm{RCP}}}{\left(\sigma_{\mathrm{ext}}^{\mathrm{LCP}} + \sigma_{\mathrm{ext}}^{\mathrm{RCP}}\right)/2} \tag{S4}$$

Proceeding into the growth algorithm proper, we extract the mesh of the NC's surface and the values of $Rate_{\mathrm{HE}}(\mathbf{r})$ evaluated at each of its points. We will then deform the mesh using the following procedure, which we implement in Matlab code. We first perform the necessary data averaging to include results from different light propagation directions, if working in *colloidal* illumination conditions. Once we have the averaged values of $Rate_{\mathrm{HE}}(\mathbf{r})$, each point of the mesh defining the surface of the gold NC will be displaced—along the direction of the surface normal at that point—by a distance dependent on the value of $Rate_{\mathrm{HE}}(\mathbf{r})$ at that point and at its neighbors. In particular, we compute the absolute growth of the *i*-th point, $G(\mathbf{r}_i)$, at position $\mathbf{r}_i$, as

$$\begin{gathered} G(\mathbf{r}_i) = \frac{1}{4}\left(Rate_{\mathrm{HE}}(\mathbf{r}_i) + \frac{3}{W} \sum_{|\mathbf{r}_j - \mathbf{r}_i| \le r_{th}} w_j Rate_{\mathrm{HE}}(\mathbf{r}_j) \right) \\ W = \sum_{|\mathbf{r}_j - \mathbf{r}_i| \le r_{th}} w_j; \qquad w_j = \frac{1}{|\mathbf{r}_j - \mathbf{r}_i|} \end{gathered} \tag{S5}$$

This is a straightforward weighted average, which implements the hypothesis that the growth will be impacted not only by the HE excited at that very point, but also by those excited up to a distance $r_{th} = 2\ \mathrm{nm}$, even though weighted by the inverse of their distance to the point under evaluation. In the case of the Au@PbO_2 prism, This procedure creates a surface map of the growth that effectively smooths out some of the numerical noise in $Rate_{\mathrm{HE}}(\mathbf{r})$, arising from the finite precision of the simulation and the mesh discretization. Finally, the displacement of each point in the mesh, d_i, will be determined by these growth maps, as

$$d_i = D_{\mathrm{t}} \frac{G(\mathbf{r}_i)}{\max\left[G(\mathbf{r}_i)\right]} \tag{S6}$$

where D_{t} is a parameter fixing the maximum mesh growth per step. By defining d_i in this relative way, the interval of time elapsed in each growth step will be in general different between steps and geometries, as what we are fixing is the maximum growth per step. This is convenient because we can limit the amount of deformation before we compute each new optical response of the NC. It also abstracts the kinetics of the specific reaction driving the photogrowth and allows us to focus instead on the sensitivity of the chiral growth to NC

geometry. We have chosen small values of D_t to approximate the real evolution of the system, in which we can expect the growth to proceed by small—atomic—increments, thus quasi-continuously. Finally, after deforming the mesh we perform a Laplacian mesh smoothing[14] to avoid the creation of sharp peaks resulting from strong gradients in d_i between mesh points, as these would create small but intense spurious hot spots. Such scenario would result from the nonlinear amplification, over several growth steps, of variations such as numerical noise due to the finite precision of the calculations. After this smoothing step, we arrive at a mesh that configures the final geometry of the NC after the given growth step, and which will be used to compute both the spectral response of this growth step and the $Rate_{HE}(\mathbf{r})$ maps that will inform the following growth step.

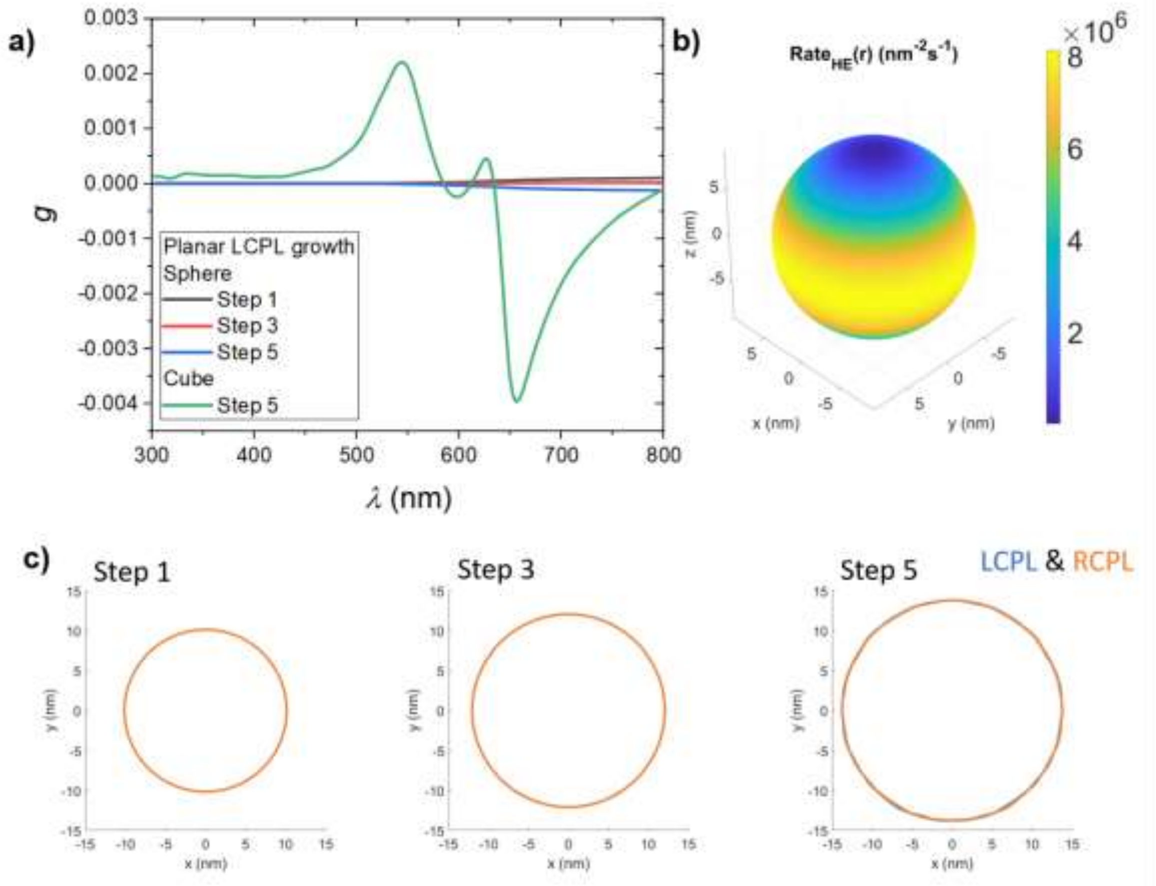

Fig. S1: Growth of small spherical nanoparticle under right-handed circularly polarized light. a) Plot of the dissymmetry factor g after several steps of growth under planar illumination conditions. We present the final spectrum for the cube to provide a point of comparison. b) Initial map of the rate of generation of hot carriers before the first growth step. c) Cross sections of the two enantiomers at the plane $z = 0$ for the three growth steps corresponding to the data in panel a. There is an almost perfect overlap between both datasets.

Rotational symmetry of the NCs

When we move from a NC with perfect rotational symmetry to one with reduced rotational symmetry, the latter offers special points in their optical response that can serve as seeds for chiral growth under CPL illumination (Fig. S2a). In studying geometries with a broken rotational symmetry, we will contrast NCs with cross sectional profiles belonging to different symmetry groups. We have considered five polyhedra with planar cross sections belonging to the C_{1v}, C_{2v}, C_{3v}, C_{4v}, and C_{5v} point groups, contextualizing the cube within NCs progressively departing from continuous rotational symmetry. It is interesting to first focus on the three geometries corresponding to the last point groups mentioned—i.e. the triangular prism, the cube and the pentagonal prism, respectively. They have a similar volume, so that their fundamental differences are mainly those arising from their different symmetry. One of such differences is the angle subtended between each of their lateral faces, and its immediate consequence over the optical response of the NCs. The sharper these edges are, the stronger the hot spots will be, and thus a higher maximum of $Rate_{\mathrm{HE}}(\mathbf{r})$. A second difference is the total number of hot spots present in the NC, which increases with the number of faces of the NC. From this, we expect a higher number of developed features for the pentagonal prism in comparison with both cube and triangular prism. In Fig. 4b we can see $\Delta Rate_{\mathrm{HE}}(\mathbf{r})$ maps for these geometries, which we can contrast with that of the rectangular prism—having a C_{2v} point group symmetry due its longer side—in Fig. 4c. Instead of seeing opposite signs in $\Delta Rate_{\mathrm{HE}}(\mathbf{r})$ at the sides of each vertical edge, in the rectangular prism this sign difference extends across the vertical faces. This feature creates the seed for a more robust photogrown chirality, because the resulting chirality will not depend solely on the direction of growth of the metal at each hot spot, as it was the case for the previous three geometries, but instead the chiral features develop at distinct sites of the geometry. This, together with maximum values in $\Delta Rate_{\mathrm{HE}}(\mathbf{r})$ larger by one order of magnitude, points to the large comparative advantage of using NCs with aspect ratios above one as non-chiral seeds for chiral photogrowth. Further demonstration of this idea can be found in Fig. 4d, showcasing a prism with a cross section in the shape of an isosceles triangle and a corresponding C_{1v} symmetry. In contrast with the equilateral triangle in Fig. 4b, having a longer side separates two of the corners as being the main loci for the excitation of hot carriers, and its reduced symmetry allows the CPL illumination to excite preferentially only one of these two. Thus, like the rectangular prism, this geometry can provide chiral photogrown features that are more robust to different sources of noise in the process. Also, studying this triangle has interesting implications for realistic samples of triangular prisms with equilateral cross sections. From these results we can see that polydispersity in a sample of nominally equilateral triangular prisms can significantly change the fundamental growth symmetry in these type of chiral photogrowth.

When it comes to geometries resulting of growing the triangular, square, and pentagonal seeds, we can appreciate a relatively homogeneous trend. Firstly, and as seen in Fig. S2, they share in common that the chiral photogrowth differentiation occurs in all their corners. Then, in Fig. 4d-f, we can also see how decreasing the rotational symmetry of the NC slightly increases the size of the features grown at the corners of the system. We should remember that we are fixing the growth evolution per step through the maximum deformation length (see Computational Methods above), so that these differences mostly affect the width and laterality of these hot-spot features, not their overall length. Interestingly, from Fig. 4c we can see that their chiroptical signals are qualitatively similar. The amplitudes of their CD_{ext} peaks increase with the increasing number of corners in the seed geometry. The same picture is preserved when evaluating their *g*-factors (Fig. S7). We can also see that there is a slight blue-shift in the same direction, with increasing number of corners, but this is also connected with the position of the plasmonic peak of the non-chiral seed (Fig. S8). It is also interesting to note that the predicted chiral photogrown shapes for the triangular prism are quite similar to those resulting from the growth of Au nanotriangles in presence of L/D-cysteine. [15]

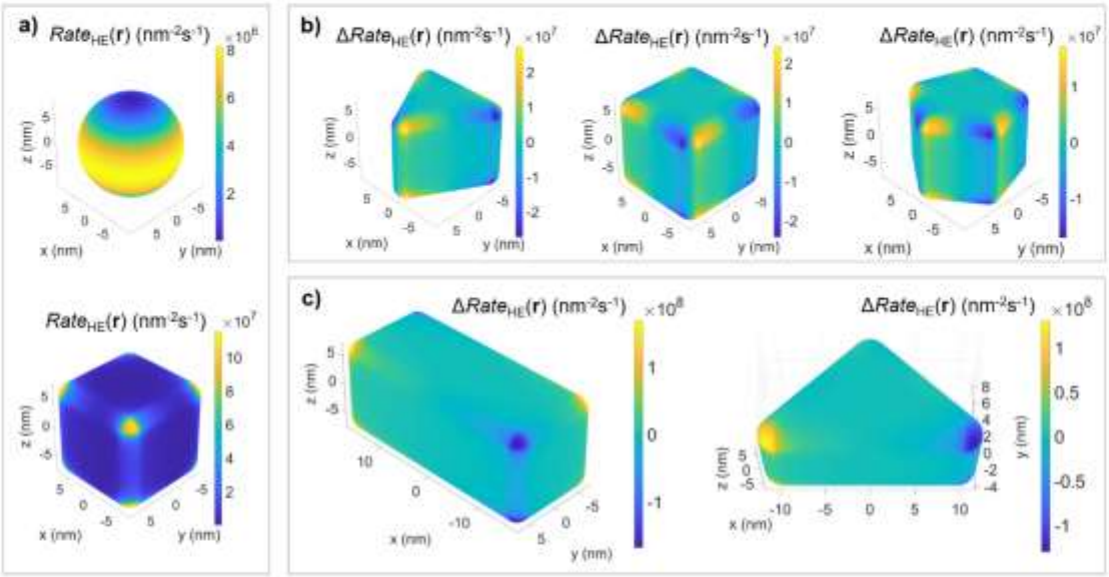

Fig. S2: Initial $Rate_{\mathrm{HE}}(\mathbf{r})$ maps for small non-chiral Au geometries under planar illumination conditions, immediately prior to the first growth step. Each NC is illuminated at its main plasmonic resonance (see Fig. S7). a) Sphere and cube under planar LCPL. The rotational symmetry of the sphere does not produce any hot spot, while the sharper features of the cube do. b) Maps of $\Delta Rate_{\mathrm{HE}}(\mathbf{r})$ for geometries with sharp features inducing an asymmetry between left- and right-CPL, and with increasing degree of rotational symmetry, ordered from left to right. c) Increasing the aspect ratio of the geometries above one, i.e. elongating the particles, creates a much stronger asymmetry between left- and right-CPL, which can be seen clearly appreciated in these $\Delta Rate_{\mathrm{HE}}(\mathbf{r})$ maps, with each polarization preferentially exciting a set of corners of the prism, or one of the acute angles in the isosceles triangular prism. Also note the order of magnitude difference with respect to data in panel b.

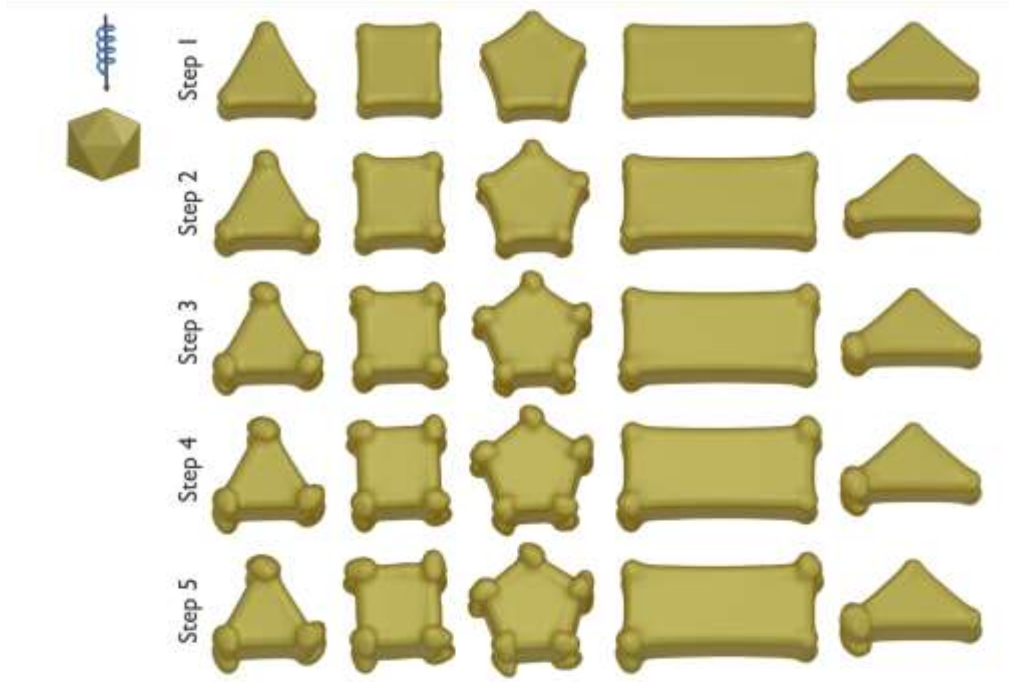

Figure S3: Evolution of the shape of five non-chiral Au NC geometries for consecutive steps of photogrowth under planar illumination conditions. All of them showcase a progressive increase in their chirality as the nonlinear photogrowth proceeds.

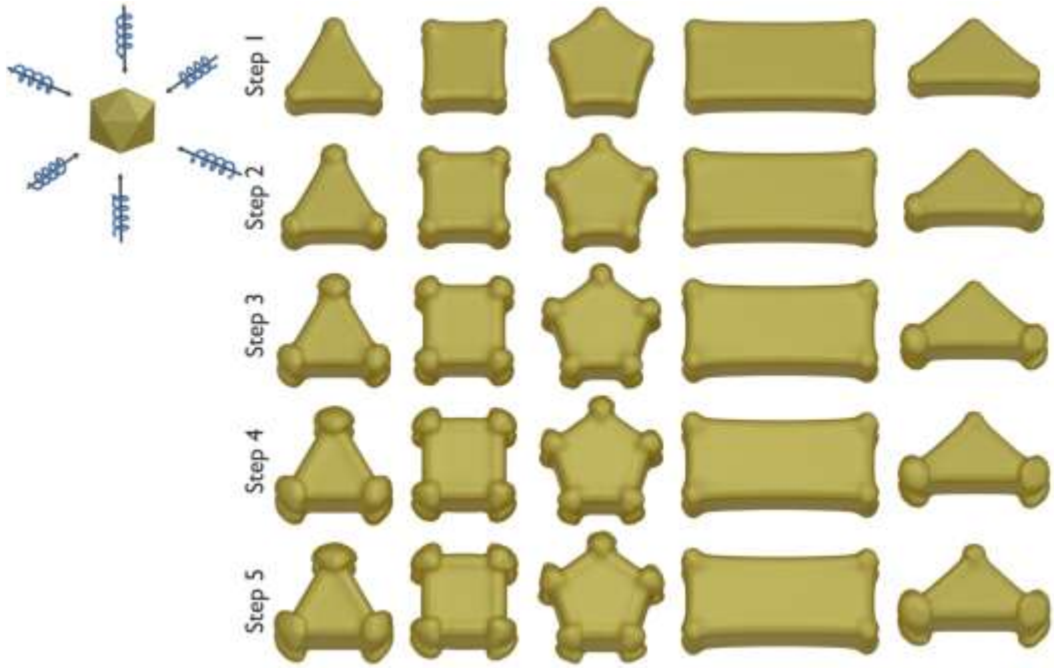

Figure S4: Evolution of the shape of five non-chiral Au NC geometries for consecutive steps of photogrowth under colloidal illumination conditions. In the case of photogrowth under colloidal conditions, the change in geometry does not develop chiral features.

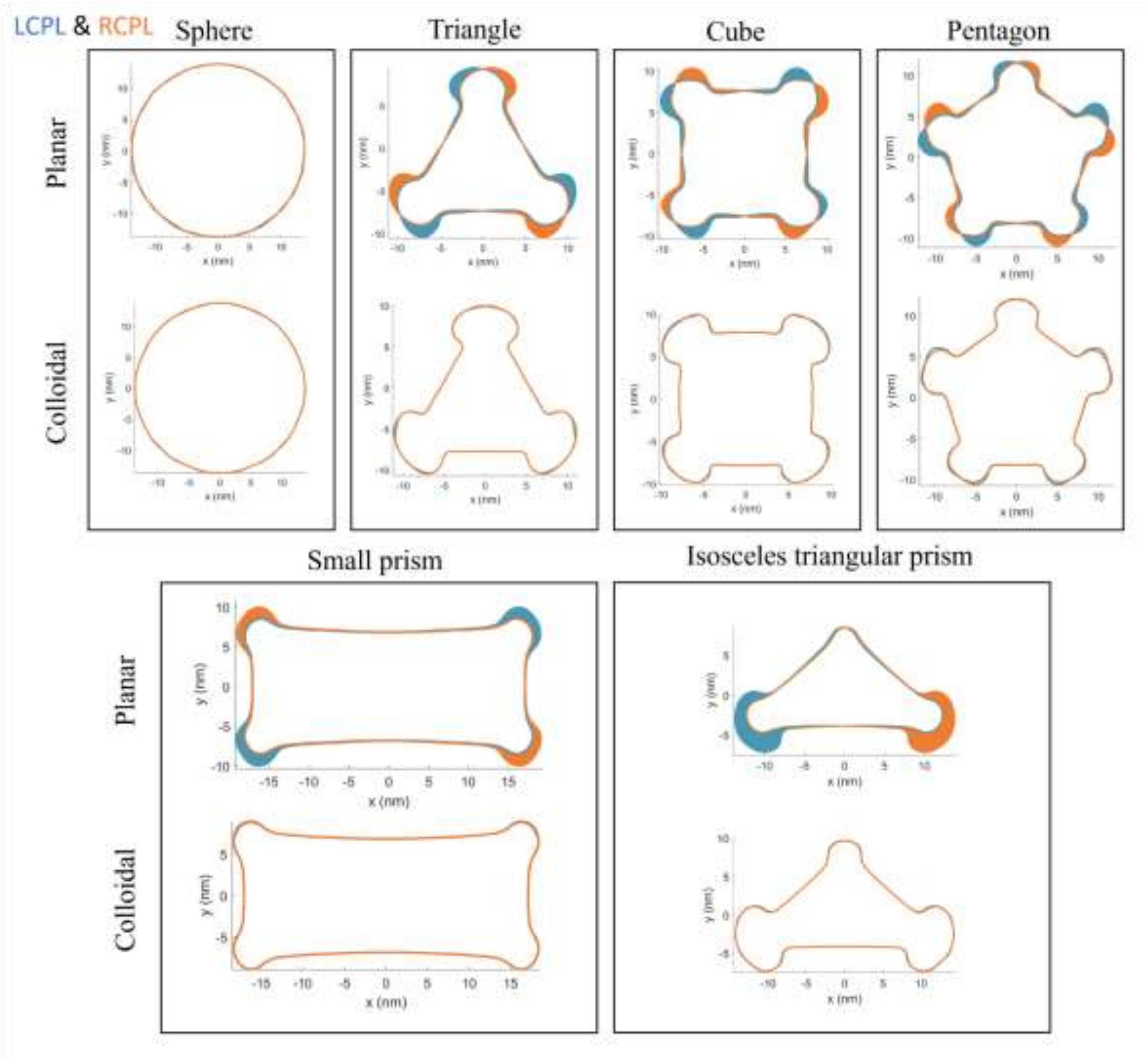

Figure S5: Cross-sectional cuts of the two enantiomers of the Au NCs after five growth steps under both types of planar illumination. The spheres are cut by their central plane, while the rest are cut at a plane 7 nm over their centers. When appreciable, the non-overlapping areas are colored to highlight the chiral symmetry of the enantiomers. As discussed in the text, we only observe chiral photogrowth under planar illumination for geometries without full rotational symmetry.

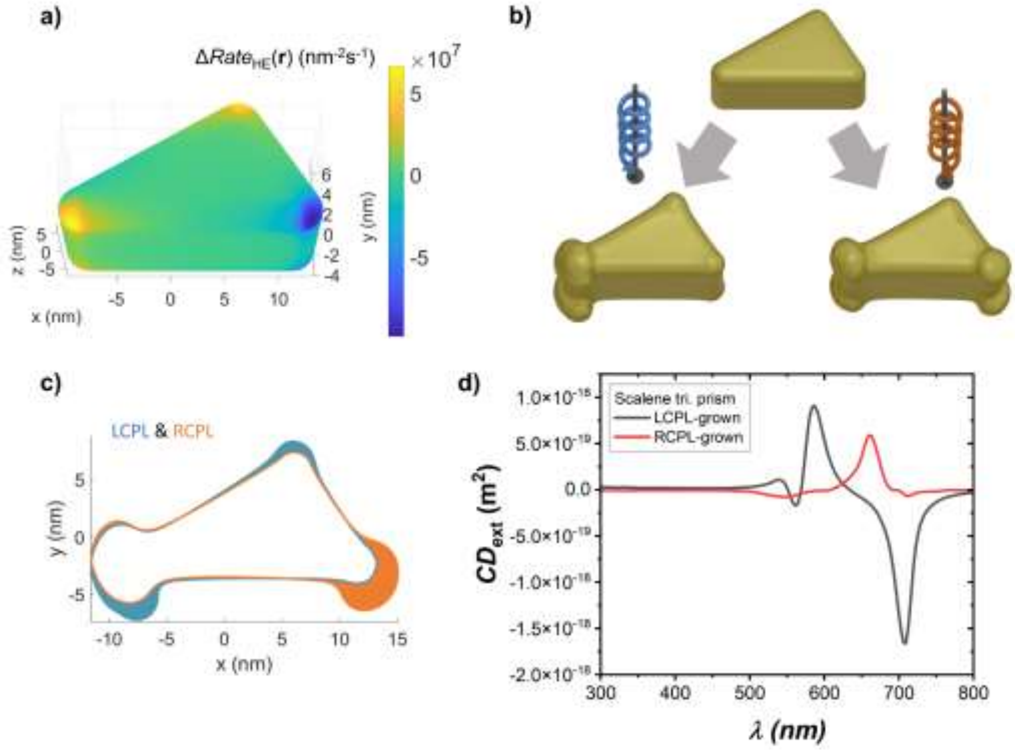

Figure S6: Chiral photogrowth of prism with a scalene triangle cross section, in planar illumination conditions. Differently to the other geometries, this seed is intrinsically chiral in 2D. Consequently, its response to the two polarizations of CPL is not antisymmetric, like in the case of non-chiral seeds. It is nonetheless an interesting case study, because irregular geometries would regularly appear in realistic polydisperse NC samples. It also serves to illustrate what occurs when evaluating the photogrowth of an intrinsically chiral NC under opposite polarizations of CPL, namely that the evolution under one of the polarizations will not be antisymmetric relative to the other. This is the case because the initial state couples in a non-symmetric way with the incoming CPL.[16] a) Initial map of $\Delta Rate_{\mathrm{HE}}(\mathbf{r})$. b) The evolution from the initial seed to the resulting geometries after 5 photogrowth steps under LCPL (left) and RCPL (right). c) Cross-sectional cuts of these two resulting NCs, at 7 nm over their centers. The non-overlapping areas are colored. d) Circular dichroism spectra for both resulting NCs.

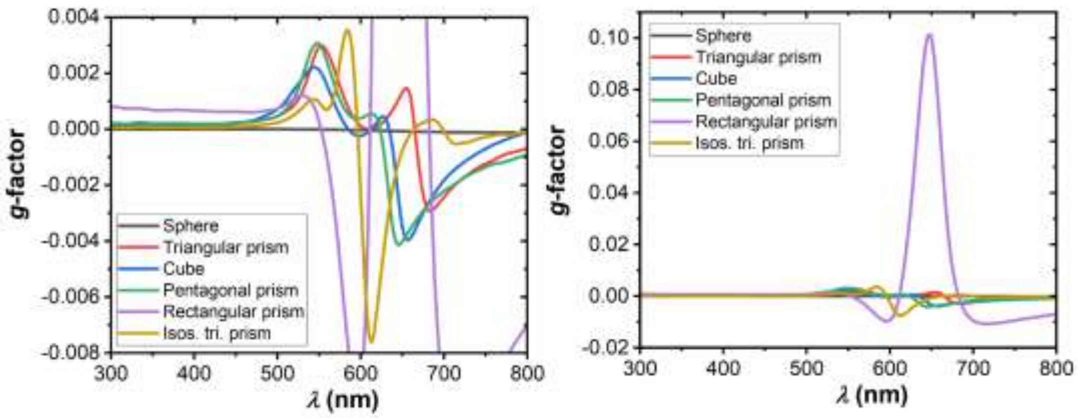

Figure S7: Dissymmetry factor for the sphere and the different Au NCs presented in Figs. 4 and 5 in the main text, after 5 steps of growth under planar illumination conditions with LCPL. The data in the panels is the same, presented at different scales to appreciate the characteristics of all spectra.

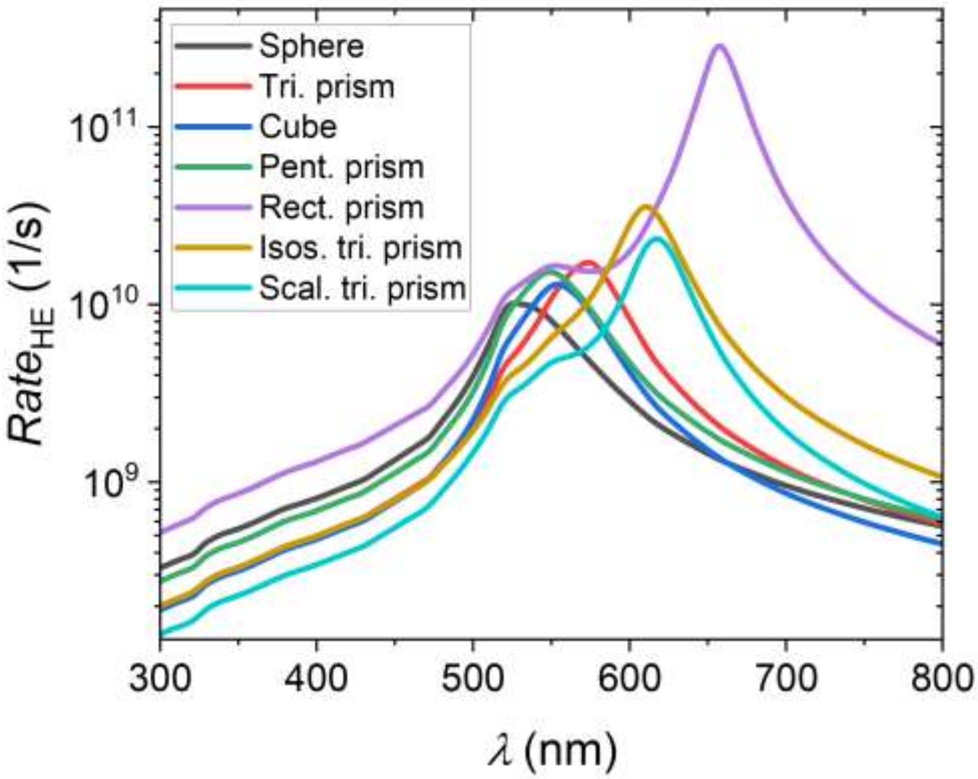

System	Growth wavelength, λ (nm)	Max growth, D_t (nm)
Au sphere	530	1
Au triangular prism	575	1
Au cube	550	1
Au pentagonal prism	550	1
Au rectangular prism	660	1
Au isosceles tri. prism	610	1
Au scalene tri. prism	620	1
Au@PbO_2 prism	550	4.5

Figure S8: Spectra for the rates of hot electron generation, under planar LCPL and before growth, for the small pure Au seeds discussed in the main text and SI. The table lists the parameters used to control the growth of each geometry. The growth wavelengths were chosen at the main peak of $Rate_{HE}(\lambda)$ for each geometry, before growth, except for the last case, which was taken to mimic the conditions in Ref. [17]. Likewise, a larger parameter D_t was chosen because, due to its larger size, larger absolute deformations caused comparatively small changes in optical response.

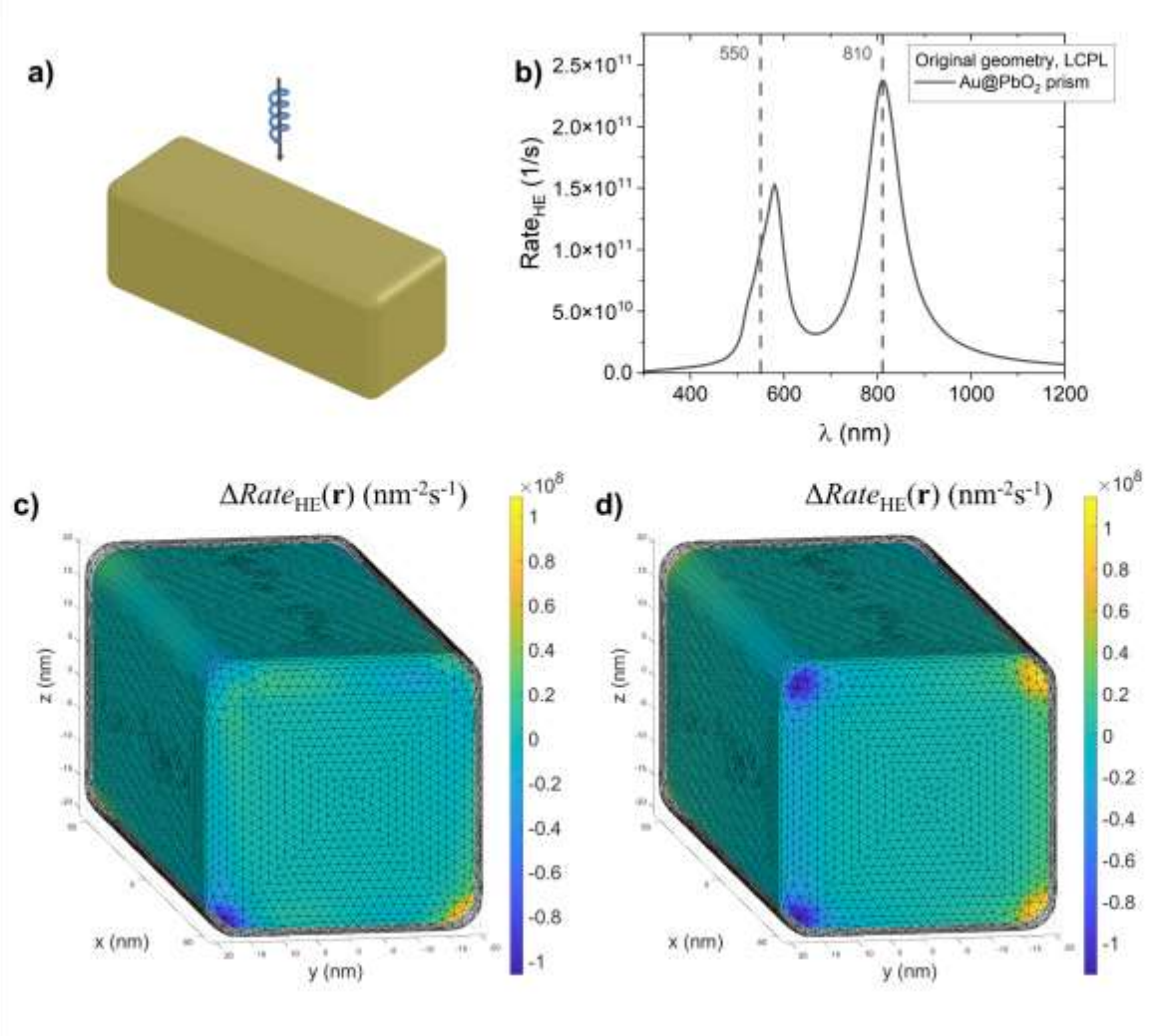

Figure S9: Initial response of the Au@PbO_2 rectangular prism. a) Diagram of the illumination conditions. b) Total $Rate_{HE}$ integrated at the interface between Au and PbO_2, illuminated under LCPL light, highlighting the wavelength under which we conducted the growth, following the setup in Ref. [17], and the peak occurring at the main plasmonic resonance. c,d) Maps of $\Delta Rate_{HE}(\mathbf{r})$ at the two wavelengths highlighted in panel b. The mesh of the PbO_2 layer is overlaid over the Ag-PbO_2 interface surface maps. It is interesting to note that the local symmetry of these modes differs mainly on the number of corners involved in the pattern of the chiral response. Exciting the prism at the high-energy mode excites primarily corners at the bottom of the NC, while the low-energy plasmon also develops strong hot spots on the top surface.

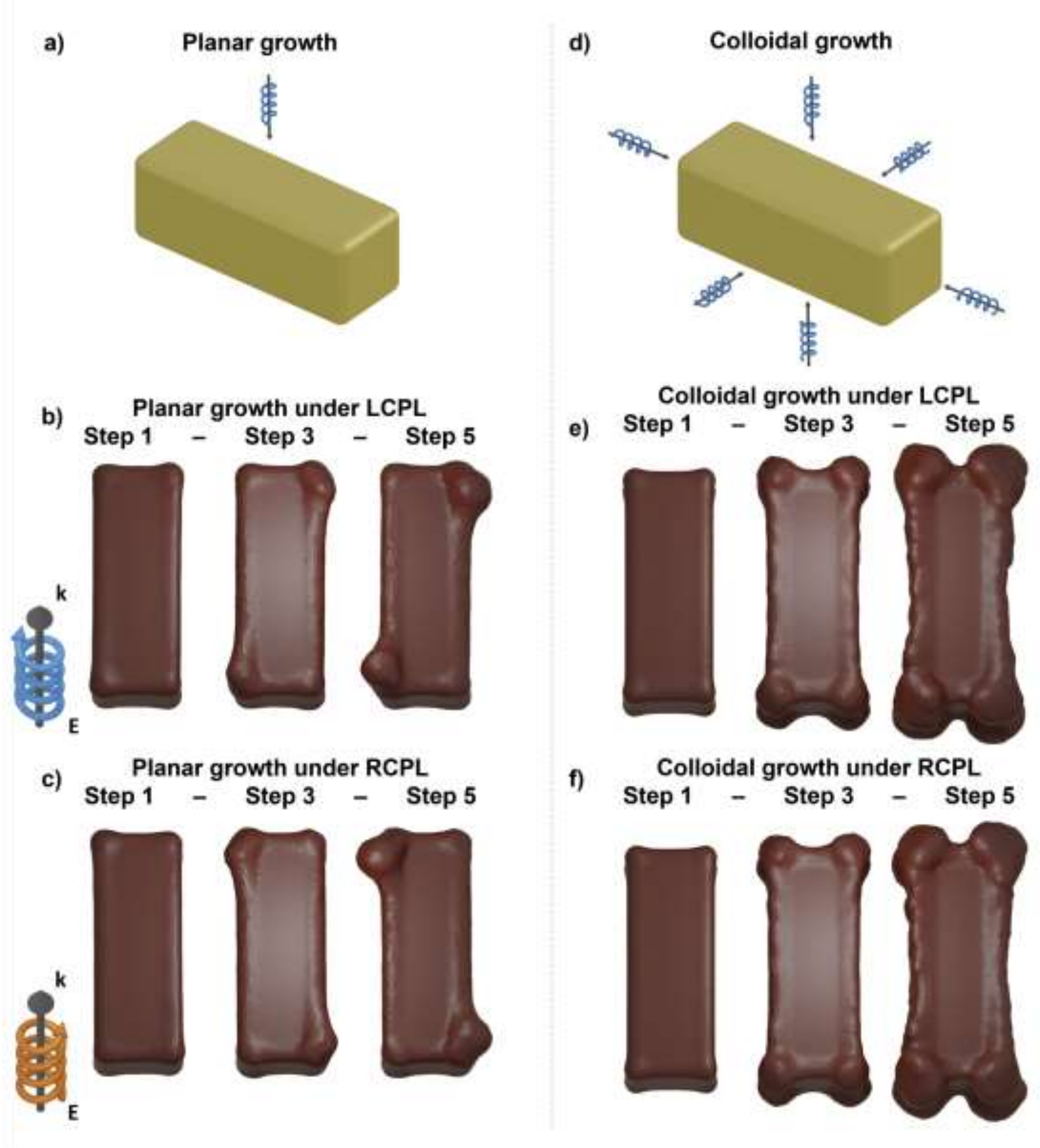

Fig S10: Evolution of the geometry of the external PbO_2 during photogrowth under the two polarizations of CPL, in both illumination conditions. Chiral features arise only under planar illumination conditions. All NCs are seen from their bottom parts, to better showcase the lumps growing under planar conditions.

References

(1) Govorov, A. O.; Richardson, H. H. Generating Heat with Metal Nanoparticles. *Nano Today* **2007**, *2* (1), 30–38.

(2) Baffou, G.; Rigneault, H. Femtosecond-Pulsed Optical Heating of Gold Nanoparticles. *Phys. Rev. B* **2011**, 13.

(3) Zhai, Y.; DuChene, J. S.; Wang, Y.-C.; Qiu, J.; Johnston-Peck, A. C.; You, B.; Guo, W.; DiCiaccio, B.; Qian, K.; Zhao, E. W.; Ooi, F.; Hu, D.; Su, D.; Stach, E. A.; et al. Polyvinylpyrrolidone-Induced Anisotropic Growth of Gold Nanoprisms in Plasmon-Driven Synthesis. *Nat. Mater.* **2016**, *15* (8), 889–895.

(4) Khurgin, J. B. Hot Carriers Generated by Plasmons: Where Are They Generated and Where Do They Go from There? *Faraday Discuss.* **2019**, *214*, 35–58.

(5) Narang, P.; Sundararaman, R.; Atwater, H. A. Plasmonic Hot Carrier Dynamics in Solid-State and Chemical Systems for Energy Conversion. *Nanophotonics* **2016**, *5* (1), 96.

(6) Hartland, G. V.; Besteiro, L. V.; Johns, P.; Govorov, A. O. What's so Hot about Electrons in Metal Nanoparticles? *ACS Energy Lett.* **2017**, *2* (7), 1641–1653.

(7) Besteiro, L. V.; Kong, X.-T.; Wang, Z.; Hartland, G.; Govorov, A. O. Understanding Hot-Electron Generation and Plasmon Relaxation in Metal Nanocrystals: Quantum and Classical Mechanisms. *ACS Photonics* **2017**, *4* (11), 2759–2781.

(8) Besteiro, L. V.; Yu, P.; Wang, Z.; Holleitner, A. W.; Hartland, G. V.; Wiederrecht, G. P.; Govorov, A. O. The Fast and the Furious: Ultrafast Hot Electrons in Plasmonic Metastructures. Size and Structure Matter. *Nano Today* **2019**, *27*, 120–145.

(9) Chang, L.; Besteiro, L. V.; Sun, J.; Santiago, E. Y.; Gray, S. K.; Wang, Z.; Govorov, A. O. Electronic Structure of the Plasmons in Metal Nanocrystals: Fundamental Limitations for the Energy Efficiency of Hot Electron Generation. *ACS Energy Lett.* **2019**, *4* (10), 2552–2568.

(10) Khurgin, J. B. Fundamental Limits of Hot Carrier Injection from Metal in Nanoplasmonics. *Nanophotonics* **2020**, *9* (2), 453–471.

(11) Sousa-Castillo, A.; Comesaña-Hermo, M.; Rodríguez-González, B.; Pérez-Lorenzo, M.; Wang, Z.; Kong, X.-T.; Govorov, A. O.; Correa-Duarte, M. A. Boosting Hot Electron-Driven Photocatalysis through Anisotropic Plasmonic Nanoparticles with Hot Spots in Au–TiO_2 Nanoarchitectures. *J. Phys. Chem. C* **2016**, *120* (21), 11690–11699.

(12) Santiago, E. Y.; Besteiro, L. V.; Kong, X.-T.; Correa-Duarte, M. A.; Wang, Z.; Govorov, A. O. Efficiency of Hot-Electron Generation in Plasmonic Nanocrystals with Complex Shapes: Surface-Induced Scattering, Hot Spots, and Interband Transitions. *ACS Photonics* **2020**, *7* (10), 2807–2824.

(13) Negrín-Montecelo, Y.; Comesaña-Hermo, M.; Khorashad, L. K.; Sousa-Castillo, A.; Wang, Z.; Pérez-Lorenzo, M.; Liedl, T.; Govorov, A. O.; Correa-Duarte, M. A. Photophysical Effects behind the Efficiency of Hot Electron Injection in Plasmon-Assisted Catalysis: The Joint Role of Morphology and Composition. *ACS Energy Lett.* **2020**, *5* (2), 395–402.

(14) Vollmer, J.; Mencl, R.; Muller, H. Improved Laplacian Smoothing of Noisy Surface Meshes. *Comput. Graph. Forum* **1999**, *18* (3), 131–138.

(15) Ma, Y.; Cao, Z.; Hao, J.; Zhou, J.; Yang, Z.; Yang, Y.; Wei, J. Controlled Synthesis of Au Chiral Propellers from Seeded Growth of Au Nanoplates for Chiral Differentiation of Biomolecules. *J. Phys. Chem. C* **2020**, *124* (44), 24306–24314.

(16) Khorashad, L. K.; Besteiro, L. V.; Correa-Duarte, M. A.; Burger, S.; Wang, Z. M.; Govorov, A. O. Hot Electrons Generated in Chiral Plasmonic Nanocrystals as a Mechanism for Surface Photochemistry and Chiral Growth. *J. Am. Chem. Soc.* **2020**, *142* (9), 4193–4205.

(17) Saito, K.; Tatsuma, T. Chiral Plasmonic Nanostructures Fabricated by Circularly Polarized Light. *Nano Lett.* **2018**, *18* (5), 3209–3212.
